# Measuring self-reliance among refugee and internally displaced households: the development of an index in humanitarian settings

**DOI:** 10.1186/s13031-021-00389-y

**Published:** 2021-07-10

**Authors:** Ilana Seff, Kellie Leeson, Lindsay Stark

**Affiliations:** 1 Brown School, Washington University in St. Louis, St. Louis, USA; 2 Independent consultant, New York, USA

**Keywords:** Self-reliance, Refugees, Internally displaced peoples, Measurement

## Abstract

**Background:**

Humanitarian practitioners have recently expanded their focus from the provision of assistance only to working to ensure refugees and internally displaced peoples (IDPs) can develop sustained ‘self-reliance’. However, few tools measure self-reliance, and even fewer capture non-financial dimensions of self-reliance or measure the construct within refugee and IDP populations. To help address these gaps in measurement and provide organizations with a tool to track households’ self-reliance over time, the Self-Reliance Index (SRI) was developed. The index component of the tool comprises 12 domains of self-reliance, including housing, food, education, healthcare, health status, safety, employment, financial resources, assistance, debt, and savings, and social capital. This paper presents the methodology used to evaluate the tool’s internal consistency and scoring validity, shares the corresponding findings, and offers a practical approach for developing a culturally relevant and robust tool for humanitarian settings.

**Results:**

Data were collected from 57 and 59 refugee households in Nairobi, Kenya, and Palenque, Mexico, respectively; repeat follow-up interviews were held with 34 and 33 households in Kenya and Mexico after a period of 3 months. Cronbach’s alpha was found to be 0.66 in Kenya and 0.64 in Mexico, both of which met the a priori minimum threshold for internal consistency of 0.6. A data-driven process was used to inform the design of the scoring rubric for the SRI, prioritizing the tool’s validity such that the final score would signal useful information about a household’s overall level of self-reliance while also keeping the process as straightforward for users as possible. Final descriptive statistics and score distributions, considered alongside organizational knowledge of sample households and sensitivity analyses, suggest good score validity.

**Conclusions:**

The SRI aims to serve as an important step in measuring the complex subject of self-reliance in a comprehensive way and over time. Results suggest that, with some contextualizing for each setting, the universal tool offers a measurement approach that is feasible, reliable, and valid. By encouraging relevant stakeholders to more holistically conceptualize and measure self-reliance, the SRI also aims to promote a more cross-sector, all-inclusive approach to programming.

**Supplementary Information:**

The online version contains supplementary material available at 10.1186/s13031-021-00389-y.

## Background

In the past decade, humanitarian crises have expanded, both in terms of numbers of individuals affected as well as average duration of displacement. UNHCR has dubbed the last decade, “a decade of displacement” with nearly 80 million people displaced at the close of 2019, nearly doubling the number of displaced people from 2010 [1]. Further, because humanitarian crises have also become more protracted, people are displaced in their countries of origin and refugees are now in transit countries for extended periods of time [2]; by 2019; 78% of refugees were impacted by a protracted crisis [3]. Although refugee camps have served as a common housing solution for refugees, today only one-third of refugees globally reside in camps or settlements; approximately 60% live in urban areas [4].

In recognition of the increasingly prolonged conflicts in recent decades, the humanitarian community has expanded its approach from focusing solely on the provision of assistance to actively working to ensure resilience and self-reliance for IDPs and refugees [2, 5–7]. UNHCR defines self-reliance as the ‘social and economic ability of an individual, a household or a community to meet its essential needs in a sustainable manner and with dignity’ [8]. However, while this widely-used definition acknowledges the importance of both the economic and social resources in achieving self-reliance, assistance models that aim to improve self-reliance often focus exclusively on the financial dimensions. Betts, Omata, & Sterck (2020) recently outlined the financial dimensions of self-reliance to include the environment, assets, access to networks, markets, and public goods. In contrast, social self-reliance focuses on the well-being of refugees and IDPs, and their communities, and includes social and collective bonds, personal safety, and health and education [9]. Recent research in Kenya indicates that in some cases, refugees prioritize their social networks over economic opportunities [10] and further research suggests that refugees themselves define self-reliance to include dimensions of self-sufficiency and well-being [2, 8]. As efforts focusing solely on economic self-reliance are not always sufficient, social aspects of self-reliance have begun to receive increased attention.

The resulting stability associated with self-reliance contributes to smoother integration, relocation, and repatriation [8, 11]. Beyond economic stability, research demonstrates the positive impact self-reliance can have on refugees’ mental well-being, often countering the distress many experience from the loss of self-reliance that occurs when they first become displaced [12, 13]. In a recent study with refugees in Kenya, participants highlighted the importance of living without aid and assistance, noting that a lack of self-reliance negatively affected their self-esteem and well-being. Other study participants shared the perceived instability that accompanied relying on aid, offering that assistance is unstable because “you never know when you will be cut off” [2]. Further research shows that across similar situations and populations, refugees who are able to work have better mental health than those who are not [14].

The humanitarian community’s shift toward self-reliance comes in part in response to more traditional emergency response frameworks’ failure to adapt to the needs and realities of current refugee and IDP populations [7]. Emergency response models have cast refugees and IPDs as vulnerable and in need of assistance instead of as resilient and capable, and have simultaneously increased their dependency on aid and decreased their ability to exercise their agency and skills [5, 8]. For humanitarian actors, as the number of refugees and IDPs continues to increase, strengthening self-reliance can also decrease the cost of aid as these individuals are able to provide for themselves and become less dependent on organizational support [8, 11]. In recognition of these changing trends, in 2018 the Global Compact on Refugees was affirmed by the General Assembly and included a focus on refugee self-reliance as one of the four objectives of the compact. Several humanitarian organizations have recently highlighted self-reliance as an integral component of their work [5, 8, 10, 11, 15–17].

Despite the widespread recognition of the importance of self-reliance for those affected by emergencies, there are few tools that measure the construct, and even fewer that capture non-financial dimensions of self-reliance or measure the construct within refugee and IDP populations. Among the limited tools available, self-reliance is typically measured at the individual level to account for the fact that the majority of vocational trainings and skills building programs target individuals [10, 16]. However, evolving discourse around UNHCR’s definition of self-reliance suggests that self-reliance can and should be measured at different levels: individual, household, and community [11].

To address these gaps in measurement and provide organizations with a tool to track refugee households’ self-reliance over time, a community-of-practice comprising non-governmental organizations, government agencies, foundations, and research institutes led by RefugePoint and the Women’s Refugee Commission worked together from March 2017 to March 2020 to create and validate the Self-Reliance Index (SRI). The SRI is a universal, semi-structured tool designed to facilitate a dialogue with refugee households around 12 domains of self-reliance. It was created to (1) support organizations in screening and targeting clients for assistance, (2) inform holistic programming and referral protocols and (3) guide organizations in responsibly “graduating” clients from assistance when it is no longer needed. The SRI may serve other purposes such as assessing program effectiveness or trend analysis across contexts and populations. Further, because the SRI was developed to be universal in nature, many of the terms used in the tool are purposefully broad so that they can be operationalized by context. For example, the SRI process includes contextualizing features of a self-reliant household in each new setting before administering the tool. The SRI was developed to fill a specific gap identified by refugee-focused actors; however, organizations working with IDPs and returnees have adopted the tool to address this wider gap within the humanitarian field.

A soft launch of the SRI was conducted from September to December 2019 to evaluate internal consistency of the 12 domains and to develop a corresponding scoring rubric to provide practitioners with a valid representation of a household’s overall self-reliance. This paper presents the methodology employed, results around internal consistency and scoring validity, and offers a practical approach for developing a culturally relevant and robust tool for humanitarian settings.

## Methods

### The tool

The full SRI tool encompasses four parts; Part 1 collects biographic information on household members, Part 2 guides the interviewer through a semi-structured discussion to collect information on 12 domains of self-reliance, Part 3 allows the respondent to provide answers to open questions, and Part 4 prompts the interviewer to provide her own assessment of the household’s self-reliance (see Additional file 1 for the full SRI tool). The 12 domains in Part 2 comprise both financial and non-financial dimensions, alike, and include: housing (dwelling and rent), food security, education, healthcare, health status, safety, employment, financial resources, assistance, debt, savings, and social capital. These 12 domains are used to feed into a household’s overall SRI score, which can assume a value between 1 and 5 and serves as the primary metric for tracking self-reliance. The SRI was first developed through a process that included a review of the literature and existing tools, input from refugees, community of practice members and experts in the field [5]. This initial phase was followed by the use of an iterative process that prioritized empirically driven efforts and ensuring that question and response wordings were contextually grounded and appropriate across multiple settings. Through this process, the tool was tested and refined in conjunction with organizational partners in three sites: Irbid, Mafraq and Amman, Jordan; Nairobi, Kenya; and Palenque, Mexico, from November 2018 to May 2019.

The SRI was not designed to be implemented as a questionnaire to read verbatim, whereby an interviewer asks a question and records the respondent’s initial answer. Rather, the SRI is administered as a semi-structured conversation between the respondent and interviewer; effective administration relies on using a combination of discussion, skilled probing, direct observation, knowledge of local conditions, and any prior knowledge of the household’s circumstances. The interviewer uses the information gleaned to select from the closed answer options that accompany each domain (see Table 1 for a full list of domains, guiding questions, response options, and scoring protocols that were used for piloting data collection; the final version of the SRI can be found in Additional file 1). The tool is typically employed with one respondent in the household, though the instrument measures self-reliance at the household level, acknowledging the importance of family and community in refugee well-being. While the SRI tool comprises four parts, the analysis presented here was carried out on data collected for Part 2 of the full SRI tool.

**Table 1:** SRI Part 2: Domains, guiding questions, and response options piloted in the field

Domain #	Domain content	Guiding question	Response options	Original scoring method
1a	Housing: Housing adequacy	How would you describe your current housing situation?	1. No shelter 2. Makeshift shelter (shack, kiosk, vehicle)/ Shelter not fit for safe habitation 3. Temporarily hosted by friends, family, community/faith group, or emergency shelter 4. Apartment or house, not adequate 5. Apartment or house, adequate	Score is equal to response option number
1b	Housing: Rent	How many months in the last 3 months have you not been able to pay rent?	1. 2–3 times 2. 1 time 3. None 4. Not applicable	Response 1 = 1 2 = 3 3 = 5 4 = Unscored domain
2	Food	How would you describe your household’s food intake yesterday?	1. Household did not eat yesterday 2. Household was able to eat, but not even a full meal 3. Household was able to eat 1 full meal 4. Household was able to eat 2–3 full meals	Response 1 = 1 2 = 2 3 = 3 4 = 5
3	Education	In the last 3 months, have the school-aged children in your household been attending school?	0. No school-aged children in household 1. None are in school 2. Some are in school 3. All are in school	0 = Unscored domain 1 = 1 2 = 3 3 = 5
4	Health care	In the last 3 months, has your household been able to get the health care needed?	0. Have not needed health care in last 3 months 1. Did not receive the needed health care 2. Received some of the needed health care 3. Received all of the needed health care	0 = Unscored domain 1 = 1 2 = 3 3 = 5
5	Health status	Does anyone in your household currently have a physical or psychological health condition that interferes with income-generating activities?	1. Adult(s) in household has condition that completely prevents employment 2. Adult(s) in household has condition that restricts or temporarily prevents employment 3. Dependent(s) has health condition that completely prevents adult employment 4. Dependent(s) has health condition that restricts or temporarily prevents adult employment 5. Adult(s) or dependent(s) may or may not have a health condition, but doesn’t prevent employment	Score is equal to response option number
6	Safety	Does your household currently feel safe enough to pursue all of the social, economic and educational opportunities you want?	1. Don’t feel safe enough to pursue any opportunities 2. Feel safe enough to pursue some opportunities 3. Feel safe enough to pursue all opportunities	1 = 1 2 = 3 3 = 5
7	Employment	How would you describe the income-generating activities that household members are engaged in, in the last 3 months?	1. No employment 2. Temporary, irregular, seasonal 3. Regular part-time (including self-employment) 4. Full-time (including self-employment), without necessary legal documentation 5. Full-time (including self-employment), with legal documentation, if necessary	Score is equal to response option number
8	Financial resources	In the last 3 months, how is your household supporting itself to meet its basic needs? [select as many as apply]:	1. Assistance 2. Borrowing money 3. Selling assets 4. Previous savings 5. Remittances/money/in-kind contributions given by friends or relatives 6. Work (including formal and informal work, petty trade, handicrafts, services, etc.)	- If ‘6’ is not selected, then score is 1 - If ‘6’ is selected AND any other option is selected, then score is 3 - If ‘6’ is the only option selected, then score is 5
9	Assistance	Have you relied on assistance for any of the following in the last 3 months? [select as many as apply]:	0. No assistance 1. Food 2. Utilities/Housing 3. Healthcare 4. Education (primary and/or secondary education) 5. Other (include a description in Comments section)	3 or 4 items selected = 1 2 items = 2 1 item = 3 0 items = 5
10	Debt	Do you currently have any debt (no matter how small) for any of the following? [select as many as apply]:	0. No debt 1. Food 2. Utilities/Housing 3. Healthcare 4. Education (primary and secondary education) 5. Transport 6. Investment (include a description in Comments section)	4 or 5 items selected = 1 2 or 3 items = 2 1 item = 3 0 items = 5
11	Savings	Do you currently have any money you have saved or put aside, or assets you could sell if needed?	1. No, no savings or sellable assets 2. Yes, but not enough to cover 1 month’s expenses (basic needs) 3. Yes, enough to cover 1 month’s expenses (basic needs) 4. Yes, enough to cover 1 month’s expenses (basic needs) plus enough to purchase an asset, or reinvest into one’s business, or to sustain a moderate health crisis	Response 1 = 1 2 = 3 3 = 4 4 = 5
12a	Social capital: Financial	If someone in your household were to have an emergency, do you know people that would be able to lend you money to cover the associated costs?	1. Knowns no one who could lend money 2. Knowns someone/has community support that could lend money	Response 1 = 1 2 = 5
12b	Social capital: Relational	Are there people that you or your household members ask for advice and/or information? Are there people that ask you or your household members for advice and/or information?	0. Neither 1. Household members ask others for advice/information ONLY 2. People ask household members for advice/information ONLY 3. Both 1 and 2	Response 0 = 1 1 = 3 2 = 3 3 = 5

### Settings and partners

Data for the present analyses were collected in two sites: Nairobi, Kenya and Palenque, Mexico. Data collection in Jordan was delayed and is thus not included in this paper.

Kenya is home to nearly 500,000 refugees and asylum-seekers, with approximately 80,000 living in urban areas [18]. More than half of refugees in Kenya originate from Somalia, with others coming from neighboring countries including South Sudan, the Democratic Republic of Congo, and Ethiopia. While Kenya became a pilot country for a Comprehensive Refugee Response Framework in 2017, the government maintains an encampment policy with refugees restricted to one of the remote refugee camps on the borders of South Sudan and Somalia [19, 20]. Refugees living outside of the camps do so with special passes or illegally, creating opportunities for exploitation and abuse [20, 21]. Data in Nairobi were collected by RefugePoint, an organization that has been working to find durable and sustainable solutions for refugees since 2005. At the time of data collection, the majority of RefugePoint clients had been in Nairobi for at least a few years.

By the end of 2019, there were approximately 98,000 refugees and asylum-seekers in Mexico [22]. Approximately 60% of asylum applications in 2019 were filed by Hondurans and El Salvadorans. Mexico serves as a country of refuge to those interested in seeking asylum in Mexico along with the many looking to transit through to the United States [23]. With increasing numbers of refugees along with pressures from the United States, those seeking asylum face greater barriers to assistance, longer wait times and further exploitation [24]. Data in Palenque, Mexico, was collected by Asylum Access, an organization that works to improve access to and the quality of asylum for refugees. At the time of SRI data collection, the majority of Asylum Access clients in Palenque had been living in Mexico for less than a few months and many clients had yet to secure housing.

### Data collection

SRI data were collected through partner organizations from September 2019 to January 2020 in both study sites. Although the SRI was designed to be universal, certain components of the tool require contextualization in order to ensure collected data reflect valid representations of self-reliance in a given setting. As such, prior to data collection in a new site, SRI interviewers work together to ensure that they have a common, applied view of what a self-reliant household might look like in their context. For example, definitions of “adequate” housing in Mexico look quite different from definitions in Kenya. Similarly, while children in Mexico are considered to be of school-age at age 4, children in Jordan are not required to attend school until they reach age 6. Cultural differences around food and eating often translated to different conceptions of a “full meal” across contexts as well. The parameters of these various terms were discussed and defined with the data collection team in each site prior to data collection.

Sample sizes were determined such that data were collected for at least 10 households per each of the 12 domains in Part 2 [25, 26]. As such, it was determined that data would be collected for 120 households per site. Following discussions with partner organizations around staff and resource availability and client base, each partner organization agreed to split the 120 households over two waves of data collections. Partners would conduct initial interviews with 60 households and then re-interview these households after a 3-month period. Initial interviews were conducted with 57 and 59 households in Kenya and Mexico, respectively. RefugePoint used the tool with all of their new clients who were identified through their community navigators. In Mexico, Asylum Access used the tool with clients receiving legal services. In general, sample households were used to providing information to these organizations and, as such, there were no refusals. As a result of competing programmatic resources and the transience of clients, follow-up interviews were administered to 34 and 33 of these original households. Interviews in Kenya were administered by RefugePoint staff in September and October 2019 (baseline) and December and January (follow-up) using CommCare. Interviews were conducted in clients’ homes. Asylum Access utilized pen and paper and transferred data into excel; initial interviews were conducted in September and follow-ups in December 2019. All Asylum Access interviews took place in the organization’s Palenque offices.

The majority of data collectors received in-person training on the SRI during the development stage of the tool in the first half of 2019. The SRI development team at WRC and RefugePoint also conducted a virtual refresher training for data collectors closer to the start of data collection. The team at headquarters was also available to provide remote support throughout the data collection process. RefugePoint Kenya and Asylum Access sent data to the research team on an ongoing basis, allowing for any issues or inconsistencies in the data to be resolved in a timely fashion.

### Analysis

Following data collection, a basic scoring rubric was employed in order to assess the means, medians, and distributions of scores for each site and wave of data collection (see Table 1). In this rubric, each domain was assigned a score from 1 to 5 (where 5 signals greater self-reliance), and the final SRI score was calculated as an average of all scored domains.

#### Internal consistency

Next, internal consistency, or “the extent to which all the items in a test measure the same concept or construct” [27], was measured using Cronbach’s alpha. Alpha may take a value from 0 to 1, where a score of ‘0’ indicates that all items are independent from each other and a score approaching ‘1’ suggests that all items are highly correlated. Standard cut-offs for identifying acceptable levels of internal consistency are typically defined at 0.70 or above [28]. However, given that refugee households may receive domain-specific, targeted services, or may have experienced a shock in one or two specific domains, it is reasonable to expect that households may score differently on a few domains as compared to the rest of the domains. For example, a household may be fully self-reliant and then suddenly experience a significant neighborhood safety concern that prevents children from going to school. In this case, the household may receive a very low score on the Education and Safety domains, but a high score on all other domains. Alternatively, a household with extremely low self-reliance may have recently been targeted by a local organization for job skills training and job matching. As such, while a member of the household may now have a full-time job, the household may not yet have enough corresponding income to address issues around debt, covering basic needs, and education fees, among others. In this example, the household’s Employment score might not align with scores on the remaining domains. For this reason, the acceptable threshold was set at 0.60 and above.

Additionally, Cronbach’s alpha was estimated after removal of each domain from the set of items, in turn. For example, Cronbach’s alpha was calculated for Domains 2–12; Domains 1 and 3–12; Domains 1, 2, and 4–12; and so forth, to assess whether alpha increased when a certain domain was removed from the set [28]. Any domain for which removal resulted in a substantially increased alpha was further explored (and considered alongside findings from other analyses) to determine whether the domain was an appropriate candidate for removal or alteration. Finally, the extent to which each domain was correlated with the final SRI score was examined.

#### Scoring

The data collected in Mexico and Kenya were also used to develop and refine the scoring rubric for the 12 domains included in Part 2. Using real data to inform the scoring system helped ensure that the SRI would be valid and useful to partners across multiple country contexts. When designing the scoring rubric for the SRI, priority was given to optimizing the tool’s validity – such that the final score would signal useful information about a household’s overall level of self-reliance – while also keeping the process as straightforward for users as possible.

The final scoring rubric was adapted from an initial scoring rubric – in which the final score was the average of all domain scores (see Table 1 for domain scoring used in data collection) – keeping in mind that some domains contribute more (or less) to self-reliance than do others. For example, it was agreed that a household relying on assistance to meet all of its basic needs should not have an aggregate score on the higher end of the SRI score spectrum. Additionally, it was agreed there would be no circumstance in which a household that did not eat any food the day prior (and thus scored a ‘1’ on domain 2) would be considered highly self-reliant, but yet it is easier to imagine a scenario in which a household might not currently have strong social networks but could still be considered self-reliant overall. These qualitative distinctions implied that certain domains should contribute to the final SRI score in differentiated ways. In order to ensure that the aggregate scores reflected these considerations, the data were examined across a variety of dimensions. Specifically, the fulfillment of four conditional statements was assessed to ascertain the extent to which responses on these key domains aligned with overall scores. It was determined a priori that the scoring system should be adjusted such that these conditions were met for at least approximately 50% of relevant cases. The following conditions were assessed for domains 1, 2, 6, and 9 (Housing, Food, Safety and Assistance respectively). Findings from the internal consistency estimations, descriptive analysis, scoring conditionalities, and qualitative feedback from partner organizations, were jointly considered when constructing the final SRI scoring rubric.

## Results

A total of 91 and 92 interviews were conducted in Kenya and Mexico, respectively. Table 2 provides a summary of SRI scores as determined by the initial scoring rubric, for each country and round of data collection. Improvements in self-reliance were observed between rounds of data collection in both sites and, in average, households in Kenya exhibited greater self-reliance than those in Mexico in both rounds of data collection. The median SRI score at baseline was 3.92 in Kenya and 3.05 in Mexico, both higher than was anticipated given what was known about the interviewed households.

**Table 2:** Basic summary statistics

	Kenya	Mexico
Baseline Interviews		
Mean SRI	3.90	3.07
[SD]	[0.65]	[0.51]
Median SRI	3.92	3.05
**# of initial interviews**	**57**	**59**
Follow-up		
Mean SRI	4.29	3.72
[SD]	[0.55]	[0.63]
Median SRI	4.23	3.67
**# of follow-ups**	**34**	**33**

**Fig. 1:**
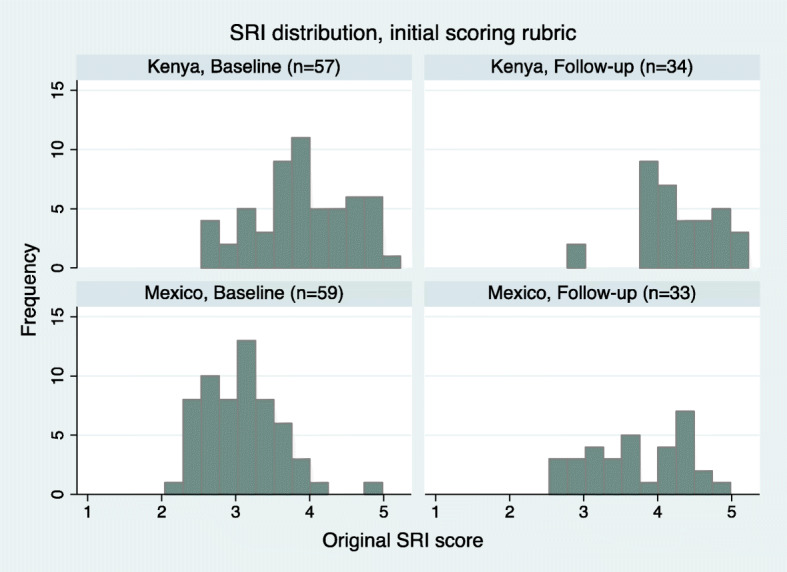
SRI distributions, initial scoring rubric

This upward skewing of the data is further demonstrated in Fig. 1, which depicts the distribution of initial SRI scores by country and round of data collection. Using the simple average of scored domains, it was found that no households in Mexico – where it was acknowledged that many interviewed households were decidedly **not** self-reliant based on practitioner observation – scored below a 2. These distributions provided an initial signal that scoring modifications were needed in order to ensure the final SRI score provided a more valid representation of a household’s overall self-reliance.

### Internal consistency

Cronbach’s alpha was calculated for the set of domains included in the SRI, combining data from both rounds of data collection within each country. Cronbach’s alpha was found to be 0.66 in Kenya and 0.64 in Mexico, both of which met the minimum threshold of 0.6. In order to identify any potentially unnecessary domains, Cronbach’s alpha was calculated an additional 12 times, each time removing one of the domains in turn. For Kenya, the removal of the Safety domain resulted in a marginally greater alpha; alpha only increased marginally with the removal of domains 5, 11, and 12 in Mexico. Finally, the extent to which each domain was correlated with the pre-adjusted SRI score was analyzed. Analysis revealed that all domains were correlated with the initial SRI score except for Domain 5 (Health Status).

The findings around internal consistency, in combination with qualitative insights from field visits and the SRI development team, were used to guide adjustments to the response options and scoring protocol for Domain 5.

### Scoring and validity

Four logical statements were tested in the data to assess the extent to which the basic scoring rubric provided a valid representation of a household’s overall self-reliance (see Table 3, Panel A). First, the data were examined to ensure that households that reported not having any housing did not score above a 3 for overall self-reliance. This condition was held in all two cases in Mexico; all households in Kenya reported having housing. Next, it was found that, among 17 households in Kenya that reported not eating a full meal the day before the interview, none met the corresponding condition of having an SRI score of 2.5 or lower; only two of 12 households met this same condition in Mexico. Similarly, none of the 15 households in Kenya that reported relying on assistance to meet almost all of their basic needs had an SRI score below 2.5, and only two of 16 households in Mexico met this condition. Finally, while no households in Kenya reported not feeling safe enough to pursue any opportunities, 12 households reported this fear in Mexico and five of those 12 had an SRI score of 3 or above. Findings from these assessments guided the decision to adjust the scoring rubric for the food and assistance domains.

**Table 3:** Conditional statements and adjusting the scoring rubric

Domain	Logical condition	Scoring condition	Panel A: Pre-SRI scoring adjustments		Decision	Panel B: Post-SRI scoring adjustments	
			Number of cases in which condition holds			Number of cases in which condition holds	
			Kenya	Mexico		Kenya	Mexico
Housing	Households that report not having any shelter should not be considered to have average or greater self-reliance.	SRI score is less than 3 for households with a domain score of 1.	N/A	2 out of 2	Acceptable	N/A	2 out of 2
Food	Households that report not eating a full meal yesterday should not be considered to have medium or high self-reliance.	SRI score is less than 2.5 for households with a domain score of 1 or 2.	**0 out of 17**	**2 out of 12**	Adjustments needed	10 out of 17	10 out of 12
Safety	Households that report not feeling safe enough to pursue any social, economic, or educational opportunities should not be considered to have average or greater self-reliance.	SRI score is less than 3 for households with a domain score of 1.	N/A	5 out of 12	Potential adjustments needed^a^	N/A	11 out of 12
Assistance	Households that report relying on assistance to meet the majority of their basic needs should not be considered to have medium or high self-reliance.	SRI score is less than 2.5 for households with a domain score of 1.	**0 out of 15**	**2 out of 16**	Adjustments needed	8 out of 15	14 out of 16

^a^Improvement in this condition was achieved through changes made to the way in which the Food and Assistance domains were integrated into the final scoring system

The final scoring rubric can be found in Additional file 2. Upon reviewing findings related to the tool’s internal consistency, distribution and domain-specific performance, along with qualitative insights provided by trainers, data collection partners and the SRI development team, changes in response options and domain scoring protocols were made to a few domains. Specifically, the SRI development team agreed that the fulfillment of three domains – Domains 2 (food), 5 (health status), and 9 (assistance) – reflected a “bare minimum” of self-reliance for these dimensions. As such, it was decided that households who did not rely on assistance, for example, should not see their overall self-reliance score increase substantially; it was agreed that these domains should only affect the final SRI score” for households that did not mean the bare minimum. Following the finalization of each domain score, the scoring rubric for the overall SRI score was modified. The final SRI score is calculated by first averaging together all scored domains, excluding domains 2, 5, and 9 (Food, Health Status, and Assistance). Subsequently, the following values are subtracted from this average as follows:

- Domain 2: Subtract (5-score2)*0.15
- Domain 5: Subtract (3-score5)*0.1
- Domain 9: Subtract (5-score9)*0.2

Finally, final scores below 1 and above 5 are recoded as 1 and 5, respectively. SRI score distributions and the logical conditions outlined in Table 3 were reassessed using the updated scoring rubric, and improvements were observed in all areas. Following these score updates, more than 50% of all domain-to-SRI scoring conditions outlined above were achieved (see Table 3, Panel B).

**Fig. 2:**
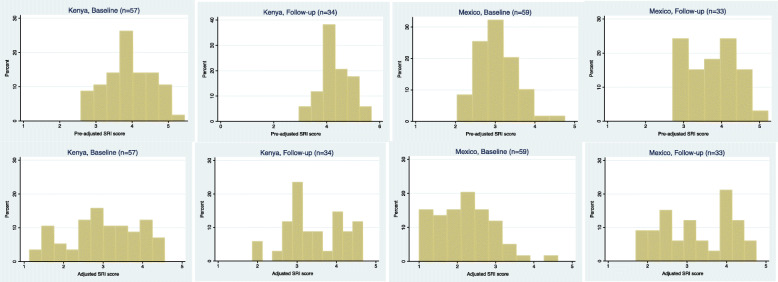
SRI score distributions, initial and updated scoring rubrics

Figure 2 compares the distributions for the original and updated SRI scores. The distribution of updated scores more closely matches what was expected of sample households given the team’s contextual knowledge and insights from the partner organizations.

### Further validation with additional data

Following the soft launch, two partner organizations collected additional data using the SRI; Caritas Switzerland collected data from 635 household clients in Syria and the Danish Refugee Council (DRC) gathered information from 120 households in Lebanon. This additional data allowed the team to perform a sensitivity analysis to test the internal consistency and scoring rubric with a larger sample size. Internal consistency was found to be marginally below adequate for Syria (alpha = 0.54) and good for Lebanon (alpha = 0.74). The conditional statements assessed in Kenya and Mexico were also examined in the data from Lebanon and Syria (see Table 4). Conditions were met in 100% of applicable cases in Lebanon and 83 to 97% of applicable cases, depending on the domain, in Syria, further validating the SRI scoring rubric. Using this scoring rubric, the average SRI scores in Syria and Lebanon were 2.45 and 2.41, respectively. Taken together, the data from Syria and Lebanon provide additional support to initial results related to internal consistency and scoring validity from the soft launch.

**Table 4:** Conditional statements and adjusting the scoring rubric

Domain	Logical condition	Scoring condition	Number of cases in which condition holds	Decision	Number of cases in which condition holds	Decision
			Lebanon		Syria	
Housing	Households that report not having any shelter should not be considered to have average or greater self-reliance.	SRI score is less than 3 for households with a domain score of 1.	N/A	N/A	N/A	N/A
Food	Households that report not eating a full meal yesterday should not be considered to have medium or high self-reliance.	SRI score is less than 2.5 for households with a domain score of 1 or 2.	8 out of 8	Acceptable	55 out of 58	Acceptable
Safety	Households that report not feeling safe enough to pursue any social, economic, or educational opportunities should not be considered to have average or greater self-reliance.	SRI score is less than 3 for households with a domain score of 1.	16 out of 16	Acceptable	172 out of 177	Acceptable
Assistance	Households that report relying on assistance to meet the majority of their basic needs should not be considered to have medium or high self-reliance.	SRI score is less than 2.5 for households with a domain score of 1.	N/A	N/A	46 out of 55	Acceptable

## Discussion

The SRI was developed to fill a gap in the humanitarian field as an increased number of the displaced live in cities and protracted situations. The challenge in developing such a tool was to create an instrument that was universal enough to be used across multiple global settings and yet still able to adequately capture the complexities and nuances in self-reliance at the local and country levels. For example, a household that did not consume food the day prior to administration of the SRI should not be considered self-reliant in any context. Similarly, a household that is reliant on formal assistance to meet all of its basic needs should not be considered self-reliant, regardless of where in the world this household resides. At the same time, the SRI must comprise response options that capture the varied representations of housing, for instance, available to refugees and IDPs across multiple urban settings in numerous countries. As such, it is of critical importance that the tool provides a valid signal as to a household’s relative self-reliance in a given setting; for this reason, the tool was designed such that it can be contextualized as needed. The empirical evidence presented here bolsters our confidence in this approach as feasible, reliable, and valid.

Our results demonstrate adequate levels of internal consistency within the two original and one of the additional study sites, suggesting that, in general, each of the 12 domains seems to capture some dimension of self-reliance across sites. Further, although the scoring rubric for the overall scores needed to be adjusted to ensure more valid representations of self-reliance within each site, the relatively higher scores in Kenya as compared to Mexico reflected what we know about the households in the two settings. While the sample households in Mexico predominantly consisted of recent arrivals, who often did not have housing or social networks in their new country, interviewed households in Kenya had typically arrived a few years prior and had well-established links to the community. These differences were reflected in the SRI scores. SRI scores for the samples from Syria and Lebanon suggested lower levels of self-reliance in these settings, which also reflects the poor quality of life for refugees and IDPs in these contexts [29, 30].

Another goal in the development of the SRI was to create an instrument to measure a complex concept in a manner that was straightforward and useful enough to be administrated by NGO staff. Humanitarian organizations on the ground often work in silos, each collecting information about – or providing services within – one area of self-reliance or well-being programming. While livelihoods program staff, for example, may be used to collecting data on household income, these teams may not normally have the opportunity to learn about the household’s feelings of safety, wellbeing, and social connectedness. Similar principles hold true for education, health, and/or psychosocial teams. The conversational nature of the SRI interview allows NGO staff to easily administer the tool while also developing rapport and building relationships with clients. The NGO teams that administered the SRI noted that the tool provided them with a more holistic picture of the household’s situation than was normally gleaned from day-to-day encounters with clients. By introducing NGOs to a holistic tool that still differs from the long, impersonal batteries of questions often administered in these settings, organizations may feel more inclined to consider their clients’ needs and opportunities in a more integrated fashion.

Finally, because the SRI and its scoring rubric have already been built into popular data platforms among humanitarian NGOs, such as Kobo and CommCare, the tool can be integrated into organizations’ existing monitoring systems. The SRI is already being used, or is in the planning stages of being used, to support program evaluations, screen for program eligibility, generate a more holistic understanding of organizations’ clients, and track the impact of policies over time. Caritas Switzerland and the DRC reported the SRI was easy to use for the first rounds of data collection presented in this manuscript and both organizations plan to continue using the tool to track these households over time.

Findings from this study can be used to help inform next steps in testing and refining the tool. Following the subjective process of improving the SRI’s validity through adjustments to the scoring rubric, future research should focus on a few areas. First, it would be useful to expand our findings to include additional country contexts. Pilot data collection was planned for sites in Kenya, Mexico, Jordan, and Ecuador, but could not be completed in Ecuador and Jordan due to administrative and security issues in-country. Future research plans for the SRI include repeating the analyses presented here for additional households and sites in order to confirm the tool’s universality. Second, there is a need for additional research to implement validity and reliability testing. Given the lack of a gold standard for measuring self-reliance in humanitarian settings, known-group comparisons offer a feasible means of assessing validity in these contexts [31] and are planned for future collections of data. The team also intends to conduct interrater and intrahousehold reliability tests to assess variability in scoring across enumerators and between household respondents. Finally, as the available sample for the SRI grows at the global level, trends in self-reliance can be tracked over time.

This study has an important limitation of note. Humanitarian settings are especially challenging given the diversity of experiences of displacement along with the ever-shifting contexts that can prove difficult for consistent training and data collection. Sample sizes for this study were significantly smaller than originally intended due to limited resources and other mobility challenges. However, additional data collected by partner organizations in Syria and Lebanon allowed us to conduct the same analyses on larger sample sizes, providing further confidence in the tool and revised scoring rubric.

## Conclusion

Recent conceptualizations of self-reliance extend beyond the unidimensional focus on economic stability at the individual level. Broader understandings of self-reliance among refugees now seek to encompass a range of domains contributing to the construct, including factors related to meeting basic needs and social capital. The SRI is intended to be a first step in attempting to measure the complex subject of self-reliance in a holistic way and over time. By encouraging relevant stakeholders to more comprehensively conceive of and *measure* self-reliance, the SRI will hopefully also spur organizations to find more innovative ways of *programming* holistically as opposed to delivering services under the current silos in which programs are often run.

## Supplementary Information

**Additional file 1.** Final SRI tool.

**Additional file 2.** Final SRI scoring rubric.

## Data Availability

Requests for data can be made to Kellie Leeson.

## Abbreviations

SRI: Self-Reliance Index
UNCHR: United Nations High Commissioner for Refugees
WRC: Women’s Refugee Commission
NGO: Non-governmental organization
