# Supplementary material for: Measuring self-reliance among refugee and internally displaced households: the development of an index in humanitarian settings
Source: Confl Health. 2021 Jul 10;15:56. doi: 10.1186/s13031-021-00389-y (PMC8272264; doi:10.1186/s13031-021-00389-y)
Supplement: Supplementary file 2 — Additional file 2. Final SRI scoring rubric. [file 13031_2021_389_MOESM2_ESM.docx]

Additional file 2. Final SRI scoring rubric

**Title:** Measuring self-reliance among refugee and internally displaced households: The development of an index in humanitarian settings

**Manuscript ID:** CONF-D-20-00165R1
**Journal:** Conflict and Health

|  | **Reviewer comment** | **Response to reviewer** | ***Revised text*** |
| --- | --- | --- | --- |
| **Reviewer 1** | | | |
| 1 | However, on the issue of the adjusted vs. unadjusted scores, I still have major concerns. The authors' analysis compares how many times the SRI score is less than a threshold when households do not meet a bare minimum to be considered self-reliant. This is examined for four particular domains. For example, on the food domain, a bare minimum is that a household should have eaten a full meal the previous day. For the unadjusted scores the SRI score is rarely below the threshold. When the scores are adjusted, the SRI score is more frequently below the threshold. The authors argue that this shows the value of the adjustment.  However, as I noted in my initial review, the adjustment in effect takes a weighted sum of the domain scores, and then subtracts 2.05. This subtraction explains the lower SRI scores, and why they are below the threshold when any of the four domains fail to meet the bare minimum. But the subtraction also lowers the scores for households where the domains do meet the bare minimum. So many of them will show scores below the threshold. (If we think in terms of sensitivity and specificity, the adjusted score is improving sensitivity, but the specificity is decreased.)  A similar result would have been found if the initial score had 2.05 subtracted from it. The alleged improvement from adjusting the scores is a sleight of hand. Further, despite the authors' statement that they decided not to weight the domains (Response to reviewer, point 6), the adjusted score is indeed weighted. (I did not notice before, but the threshold for the SRI score differs for the four domains in Table 4. For housing and safety, it is 3; for food and assistance, it is 2.5. This is inappropriate; the threshold should be the same for all four domains.) | We thank the reviewer for this feedback on the SRI's scoring approach. We acknowledge that the final SRI score is not constructed in the most straight-forward, traditional manner, but hope the reviewer will respect that our approach has been largely informed by the Refugee Self-Reliance Initiative's conceptualization of self-reliance (Leeson, Slaughter, and Buscher, under review), the trajectories of refugees' well-being, and the data that is most useful for organizations serving refugees. In our conceptualization of self-reliance, each underlying domain does not contribute to self-reliance equally; perhaps stated more accurately, different domains contribute to self-reliance *in different ways*. The assistance domain offers an illustrative example. By definition, a household that receives even one form of assistance is not fully self-reliant. At the same time, *not* receiving any assistance does not make a household "extra" self-reliant, but rather, a household that does not receive any assistance has met a prerequisite condition to being *considered* as self-reliant.  Based on this conceptualization, relying on assistance will decrease the final SRI score, but *not* relying on assistance will and should not raise the SRI score. In this way, one might think of domains 2, 5, and 9 feeding into the final SRI as "adjustments." After calculating an initial score to capture part of a household's self-reliance (as represented by the average of domain scores), we then make an adjustment to this score that reflects whether or not a household has achieved the bare minimum for these three prerequisite domains.  Further, it is important to clarify that these domains *do* *not* lower a household's SRI score when they reflect the bare minimum (in this case, defined as having a score of '5'). If a household scores a 5 for domains 2, 5 and 9, nothing is subtracted from the final score. |  |
